# RNA Sequencing Demonstrates Ex Vivo Neocortical Transcriptomic Changes Induced by Epileptiform Activity in Male and Female Mice

**DOI:** 10.1523/ENEURO.0520-23.2024

**Published:** 2024-05-10

**Authors:** Alec J. Vaughan, Laura J. McMeekin, Kutter Hine, Isaac W. Stubbs, Neela K. Codadu, Simon Cockell, Jonathon T. Hill, Rita Cowell, Andrew J. Trevelyan, R. Ryley Parrish

**Affiliations:** ^1^Department of Cell Biology and Physiology, Brigham Young University, Provo, Utah 84602; ^2^Department of Neurology, University of Alabama, Birmingham, Birmingham, Alabama 35233; ^3^Newcastle University Biosciences Institute, Medical School, Newcastle upon Tyne NE2 4HH, United Kingdom; ^4^School of Biomedical, Nutritional and Sports Science, Faculty of Medical Sciences, Newcastle University, Newcastle upon Tyne NE2 4HH, United Kingdom

**Keywords:** epilepsy, gene expression, seizure, sex differences

## Abstract

Seizures are generally associated with epilepsy but may also be a symptom of many other neurological conditions. A hallmark of a seizure is the intensity of the local neuronal activation, which can drive large-scale gene transcription changes. Such changes in the transcriptional profile likely alter neuronal function, thereby contributing to the pathological process. Therefore, there is a strong clinical imperative to characterize how gene expression is changed by seizure activity. To this end, we developed a simplified ex vivo technique for studying seizure-induced transcriptional changes. We compared the RNA sequencing profile in mouse neocortical tissue with up to 3 h of epileptiform activity induced by 4-aminopyridine (4AP) relative to control brain slices not exposed to the drug. We identified over 100 genes with significantly altered expression after 4AP treatment, including multiple genes involved in MAPK, TNF, and neuroinflammatory signaling pathways, all of which have been linked to epilepsy previously. Notably, the patterns in male and female brain slices were almost identical. Various immediate early genes were among those showing the largest upregulation. The set of down-regulated genes included ones that might be expected either to increase or to decrease neuronal excitability. In summary, we found the seizure-induced transcriptional profile complex, but the changes aligned well with an analysis of published epilepsy-associated genes. We discuss how simple models may provide new angles for investigating seizure-induced transcriptional changes.

## Significance Statement

It is well-established that strong neuronal activation results in large-scale transcriptomic changes. Understanding this process is of particular importance in epilepsy, which is characterized by paroxysmal pathological discharges. However, the complexity of in vivo activity patterns present many difficulties in interpreting the transcriptional changes. In contrast, ex vivo seizure models provide better experimental control and quantification of activity patterns with lower welfare impact. Importantly, we now show that these models also replicate the transcriptional patterns previously reported in chronic human and animal epilepsy, thus validating their use in these kinds of studies.

## Introduction

It has long been established that neuronal activity has a powerful influence on gene expression (Sheng et al., 1993; Lee and Fields, 2021). In turn, altered gene expression in neurons leads to changes in neuronal function, constituting an essential form of feedback control within the brain, albeit mediated on time-scale orders of magnitude slower than synaptic interactions. These slow transcriptional feedback mechanisms are, however, likely to play an important role in epileptic pathophysiology, given that seizures represent the most intense form of neuronal activation. It is crucial, therefore, to characterize precisely how acute seizure activity alters the transcriptomic landscape (Morgan et al., 1987). Gene expression changes that reduce neuronal network excitability can be characterized as negative feedback mechanisms, serving a protective, homeostatic function. In contrast, changes that increase neuronal excitability, depending on where in the network they occur, can act as positive feedback mechanisms, leading to an escalation of the pathophysiology. Both sets of changes, however, are likely to alter the interictal functionality of the network and thus contribute to comorbidity in epilepsy, including its effects on memory, attention, and general mental health. Of course, seizures will impact the neuronal network and other critical cell types, such as glial cells. Mutations in the astrocyte population can account for increased seizure susceptibility (Djukic et al., 2007; Ohno, 2018). In addition, oligodendrocytes undergo increased myelination during seizure activity (Knowles et al., 2022b), representing another glial cell class that likely impacts ictogenesis. Microglia cells represent another important cell class implicated in epilepsy that undergo significant changes during strong cellular activation (Vezzani et al., 1999; Kinoshita and Koyama, 2021). In short, a strong imperative remains to understand seizure-induced transcriptional changes in neuronal and non-neuronal cells within the brain and to develop robust methodologies for studying these.

To address this shortfall in our understanding, many groups have undertaken genetic, epigenetic, and transcriptome studies to explore causal links that lead to epilepsy and also explore possible protective changes (Hunsberger et al., 2005; Ryley Parrish et al., 2013; Hansen et al., 2014; Pitkanen et al., 2015; T. Chen et al., 2017). These large-scale transcriptomic data may further provide indications for novel therapeutic targets or extend our understanding of the basic pathophysiology of seizures by identifying “molecular hubs” (Gorter et al., 2006; Dixit et al., 2016; Dong et al., 2020; Kane et al., 2022). Despite this breadth of research, we still have a limited handle on many of the causal effects of brain injury, genetic alterations, status epilepticus, or other recognized triggers for epileptogenesis (Pitkanen et al., 2015).

Many groups have demonstrated large-scale transcriptomic changes during seizure activity (Okamoto et al., 2010; Hawkins and Kearney, 2012; Dixit et al., 2016; Guelfi et al., 2019; O'Leary et al., 2020; Li et al., 2022), indicating what might be termed the “molecular hallmark” of epileptiform activity. These studies have mainly been conducted using in vivo animal models or postmortem human brain tissue, primarily from hippocampal or whole-brain tissue sections (Jamali et al., 2006; Pitkanen et al., 2015; L. Zhang et al., 2018; Conte et al., 2020). While these approaches are valuable options for observing transcriptomic changes in response to seizures, there are significant costs to these methods. For instance, the postmortem studies are potentially confounded by gene transcription changes during the terminal phase of life and may lack relevance for epileptogenesis in early-onset (development) epilepsy. The in vivo models, on the other hand, can mitigate this cost by using multiple epilepsy-induced animals, but these models often involve significant animal welfare impact, and the experimental control for such studies can be difficult.

To address both these shortfalls, we developed an ex vivo technique to probe for transcriptional changes in mouse neocortical tissue, comparing brain slices with pharmacologically induced seizures to control slices from the same brain which that were not exposed to the ictogenic treatment. We identified over 100 genes showing marked seizure-induced changes from the control levels and analyzed these using Gene ontology (GO), KEGG pathway enrichment, and Qiagen Ingenuity Pathway analyses. We found no sex differences, but the set of genes whose expression patterns changed showed an overrepresentation of several seizure-associated pathways, including MAPK, TNF, and neuroinflammatory signaling. These results prompted us to examine possible disease associations with our gene expression changes, and using ingenuity pathway analysis (IPA), we found that the predicted disease of our differential expression (DE) was indeed epilepsy, consistent with the experimental model.

Our results suggest that our ex vivo brain slices replicated the large-scale transcriptional changes observed following epileptiform activity in vivo while offering levels of accessibility for recording and experimental control not usually possible in vivo and certainly not in clinical tissue. This provides a highly refined approach to studying epileptic pathology, yielding multiple experimental replicates within biological individuals, while minimizing welfare issues and harm to the animals involved.

## Materials and Methods

### Availability of data

The code/software described in the paper is freely available online at https://rb.gy/spf86r. The code is available as Extended Data. The code was run on a MacBook Air (Retina, 13-inch, 2019) using the macOS 12.7.1 operating system. The RNA sequencing datasets generated and analyzed during the current study are available on the NCBI SRA website under the token PRJNA1032094.

### Ethical approval

All experimental procedures were approved by the Institutional Animal Care and Use Committee of the University of Alabama at Birmingham and the UK Home Office and Animals (Scientific Procedures) Act 1986 and approved by the Newcastle University Animal Welfare and Ethical Review Body (AWERB # 545).

### Slice preparation

Male and female C57BL/6 mice (Jackson Laboratory stock number 000664; age ∼12 weeks) were used in this study. Mice were housed in individually ventilated cages in a 12 h light/dark regime. Animals received food and water *ad libitum*. Mice were euthanized by cervical dislocation and brains removed and stored in cold cutting solution as follows (in mM): 3 MgCl_2_; 126 NaCl; 26 NaHCO_3_; 3.5 KCl; 1.26 NaH_2_PO_4_; 10 glucose. For local field potential (LFP) recordings, 450 µm horizontal sections were made containing the neocortex, entorhinal cortex, and the hippocampus using a Leica VT1000 Vibratome. Slices were then transferred to an interface holding chamber and incubated for 1–2 h at room temperature in artificial cerebrospinal fluid (aCSF) containing the following (in mM): 2 CaCl_2_; 1 MgCl_2_; 126 NaCl; 26 NaHCO_3_; 3.5 KCl; 1.26 NaH_2_PO_4_; 10 glucose.

Extracellular field recordings were performed using interface recording chambers. Slices were placed in the recording chamber perfused with modified aCSF to induce epileptiform activity [100 µM 4-aminopyrimidine (4AP); Librizzi et al., 2017]. Recordings were obtained using normal aCSF-filled 1–3 MΩ borosilicate glass microelectrodes (GC120TF-10; Harvard Apparatus) placed in deep layers of the temporal association area. Experiments were performed at 33–36°C. The solutions were perfused at the rate of 3.5 ml/min. Waveform signals were acquired using BMA-931 biopotential amplifier (Dataq Instruments), Micro 1401-3 ADC board (Cambridge Electronic Design), and Spike2 software (v7.10, Cambridge Electronic Design). Signals were sampled at 10 kHz, amplified (gain, 500), and bandpass filtered (1–3,000 Hz). The CED4001-16 Mains Pulser (Cambridge Electronic Design) was connected to the events input of CED micro 1401-3 ADC board and used to remove 50 Hz hum offline. Seizure-like events (SLEs) were visually identified with their start time as the time of occurrence of high-frequency rhythmic bursts (tonic phase) associated with high-frequency signals, and the events were considered to end when the interval between two after-discharges (clonic phase) was ≥2 s.

### Tissue collection and RNA extraction

After 3 h of recordings, the neocortex (containing the auditory and somatosensory cortex) was subdissected away from the entorhinal cortex and the hippocampus and then flash frozen with dry ice and stored at −80°C. For the RNA extraction, tissue was homogenized in TRIzol using an Omni bead ruptor homogenizer (Omni International), and RNA was isolated using the TRIzol/chloroform–isopropanol method following the manufacturer's instructions (Invitrogen) for collection of total RNA. RNA concentration and purity were determined using a Thermo Scientific NanoDrop One (Thermo Fisher Scientific). Fourteen total samples were collected, eight of which were exposed to 4AP and six controls that were treated identically except without 4AP treatment. Of the 4AP-treated slices, five were from male and three were from female mice. Likewise, six total slices from male and female brains were sequenced from the aCSF control group.

### RNA sequencing and DE analysis

Both the library prep and the sequencing were conducted by the Genomics Core Facility at Newcastle University on a Illumina NextSeq 500 (Illumina). Library prep was performed using the SmartSeq and Nextera XT kits. The sequencing was across two NextSeq 500 High-Output (150 cycle flow cells) producing 2 × 75 bp reads. All parts of the RNA sequencing analysis pipeline were conducted in R and can be found in their entirety in the .html file Extended Data Figure 1. Each of the samples were designated with their treatment followed by an assigned number and their sex (e.g., ACSF-5F) for the analysis. After sequencing, quality control was assessed using FastQC (www.bioinformatics.babraham.ac.uk/projects/fastqc/). The sequencing reads were aligned using the Rsubread package (Liao et al., 2019) to the GRCm39 assembly of the mouse genome accessed through the Ensembl genome annotation platform. A count matrix was created from this alignment, which was used to conduct the DE analysis through the DESeq2 package (Love et al., 2014). Data were analyzed using a factorial design with the factors 4AP treatment, sex, and a sex:treatment interacting factor to determine the cause of particular gene expression changes. Genes with a calculated adjusted *p* value of <0.05 and absolute log_2_ Fold Change (FC) values >0.585 (a 50% change in expression) were considered significant. Once our analysis of sex-specific differences in response to treatment revealed no significant DE genes, we focused on the impact of the 4AP treatment on DE. Hierarchical clustering was performed for a comparison of the top 30 upregulated and 10 downregulated genes based on log_2_FC and adjusted *p* value. The respective count values from the DE analysis were converted into *z*-scores and clustered and converted into a heatmap using the ComplexHeatmap package (Gu et al., 2016).

10.1523/ENEURO.0520-23.2024.d1 Extended Data 1 File containing the code used to run the analysis. Download Extended Data 1, ZIP file.

### GO and other functional enrichment analyses

To further characterize the associations between the 4AP treatment DE genes, functional analysis was performed on the list of 110 DE genes, referred to hereafter as the gene set. Enrichment of Gene Ontology (GO) Biological Processes (BP) and Kyoto Encyclopedia of Genes and Genomes (KEGG) pathway analyses was assessed using the clusterProfiler package (Yu et al., 2012). For the GO:BP analysis, the simplify function was used to filter redundant parent–child associations into singular, informative BP terms. Full GO results can be found in Extended Data Figure 4-1. The meshes package was used to access MeSH Disease annotations for the gene list to investigate the diseases that our gene set was associated with (Yu, 2018). Twenty-four brain-related diseases and disorders were selected to highlight, and the full list of 247 enriched disease categories, along with the full GO:BP and KEGG results, can be found in Extended Data Figure 4-1. Unless otherwise specified, all plots are of the top enriched categories (adjusted *p* values <0.05) and were plotted using the enrichplot package (Yu et al., 2012). To analyze transcription factor binding sites, we used the Homer software package to do an overrepresentation analysis for our gene list, and both de novo and known motifs were selected (Heinz et al., 2010). The function was set to search 1,000 bp upstream and 100 bp downstream of the transcription start of the specific gene. For a background dataset, we selected the whole genome to enable statistical analysis.

### IPA analysis

Qiagen Ingenuity Pathway analysis was carried out on our DE gene set using the log_2_FC and adjusted *p* value for each gene (Kramer et al., 2014). All DE genes mapped to the knowledge base and the *z*-score setting was used to quantify specific pathway activation status. Canonical pathway analysis was examined and extracted to identify pathways that were predicted by the algorithm to be activated or inhibited, the full list of which can be found in Extended Data Figure 5-1. Pathways with greater than log B-H adjusted *p* values of 1.3 were considered significant. Likewise, IPA Diseases and Function analysis was used to predict disease states based on our DE genes. B-H adjusted *p* values were used to test for significance.

### Statistics

**Figure 1.**
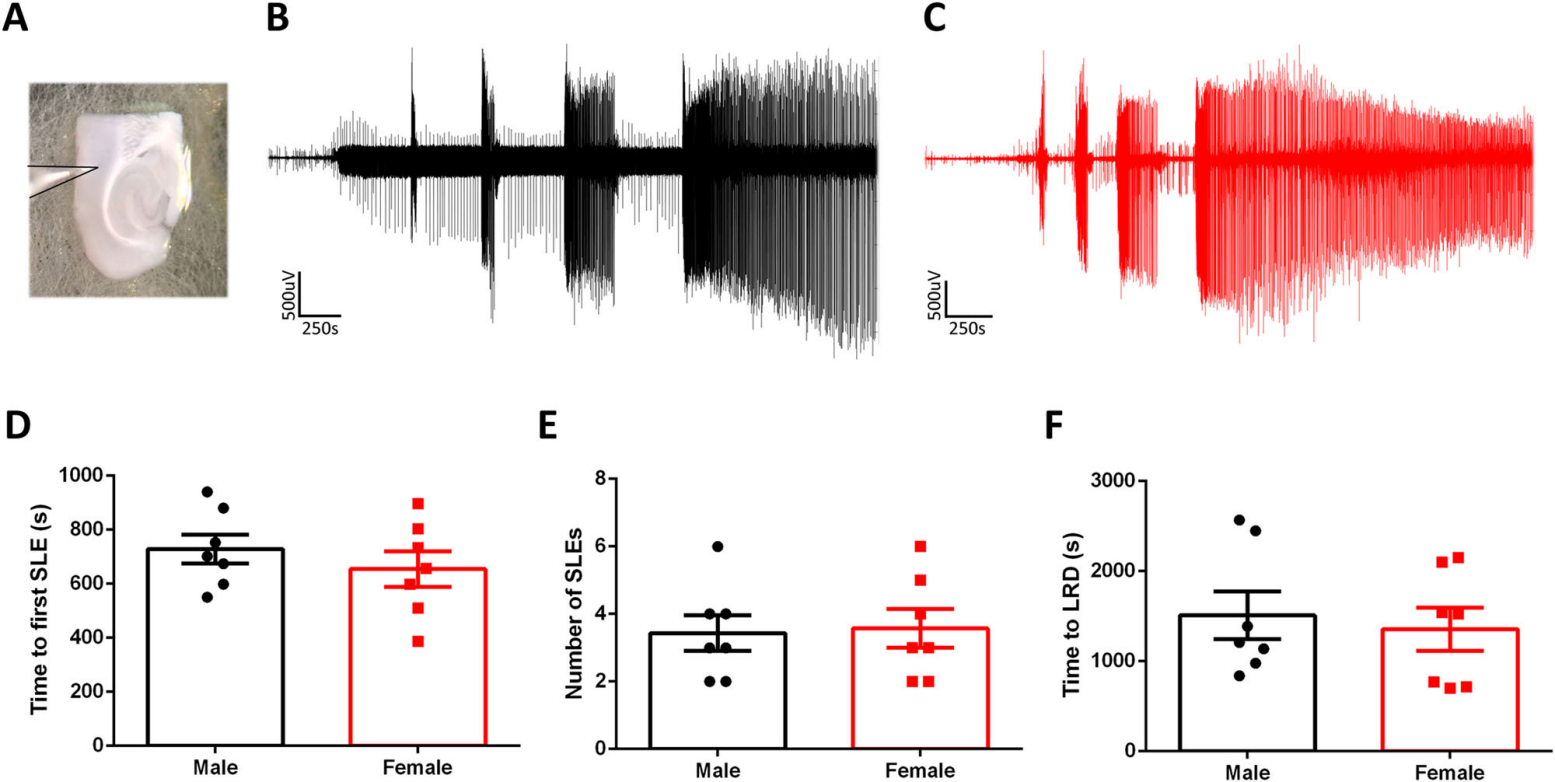
Induced seizure-like activity is similar from slices made from male and female mice. ***A***, Example of a typical neocortical deep-layer LFP recording. ***B***, Example LFP trace from a slice made from a male mouse and exposed to 4AP. ***C***, Equivalent recording from a female mouse. ***D***, There is no difference in the time to the first SLE between slices made from male and female mice (unpaired *t* test; *p* = 0.24). ***E***, There is no difference in the number of SLE between slices made from male and female mice (unpaired *t* test; *p* = 0.72). ***F***, There is no difference in the time to late recurrent discharges between male and female mice (unpaired *t* test; *p* = 0.68).

Electrophysiology data were analyzed offline using Matlab R2018b (MathWorks). Statistical tests shown in Figure 1 were analyzed using a Student's *t* tests. R was used for the analysis of all the RNA sequencing data as described above.

## Results

### Ex vivo epileptiform activity evolves in a similar pattern in both males and females

We sought to determine the utility of ex vivo seizure models for studying seizure-induced transcriptomic changes. Brain slices were prepared from male and female mice and bathed in 100 µM 4AP while recording the evolving epileptiform activity patterns (Fig. 1*A*). 4-AP induced a characteristic build-up of seizure-like activity over time, starting with early interictal-like discharges, followed by intermittent SLEs, and culminating in late recurrent discharges (Codadu et al., 2019a). This final stage appears to represent a steady state of hyperexcitability and has been suggested to be equivalent to in vivo status epilepticus (Fig. 1*B*,*C*; C. L. Zhang et al., 1995; Dreier et al., 1998).

### Ex vivo epileptiform activity shows minimal gene expression differences between the sexes

We found no difference in the evolving pattern of epileptiform activity between male and female mice (Fig. 1*D–F*), suggesting that naive susceptibility to pharmacologically induced seizure activity is similar between the sexes. We then examined whether the equivalent epileptiform activity induced distinct transcriptomic expression patterns between sexes. To address this, we conducted RNA sequencing on 14 different brain slices: 8 treated with 4AP (5 male and 3 female), all of which developed intense epileptiform discharges, and 6 control slices (3 male and 3 female), where no drug was added, and which did not display any pathological discharges. The duration of the treatment was 3 h. After this period, the RNA was isolated from the tissue. Illumina sequencing was performed on these samples, and the reads were aligned to the mouse genome for subsequent analysis of DE using the DESeq2 package.

**Figure 2.**
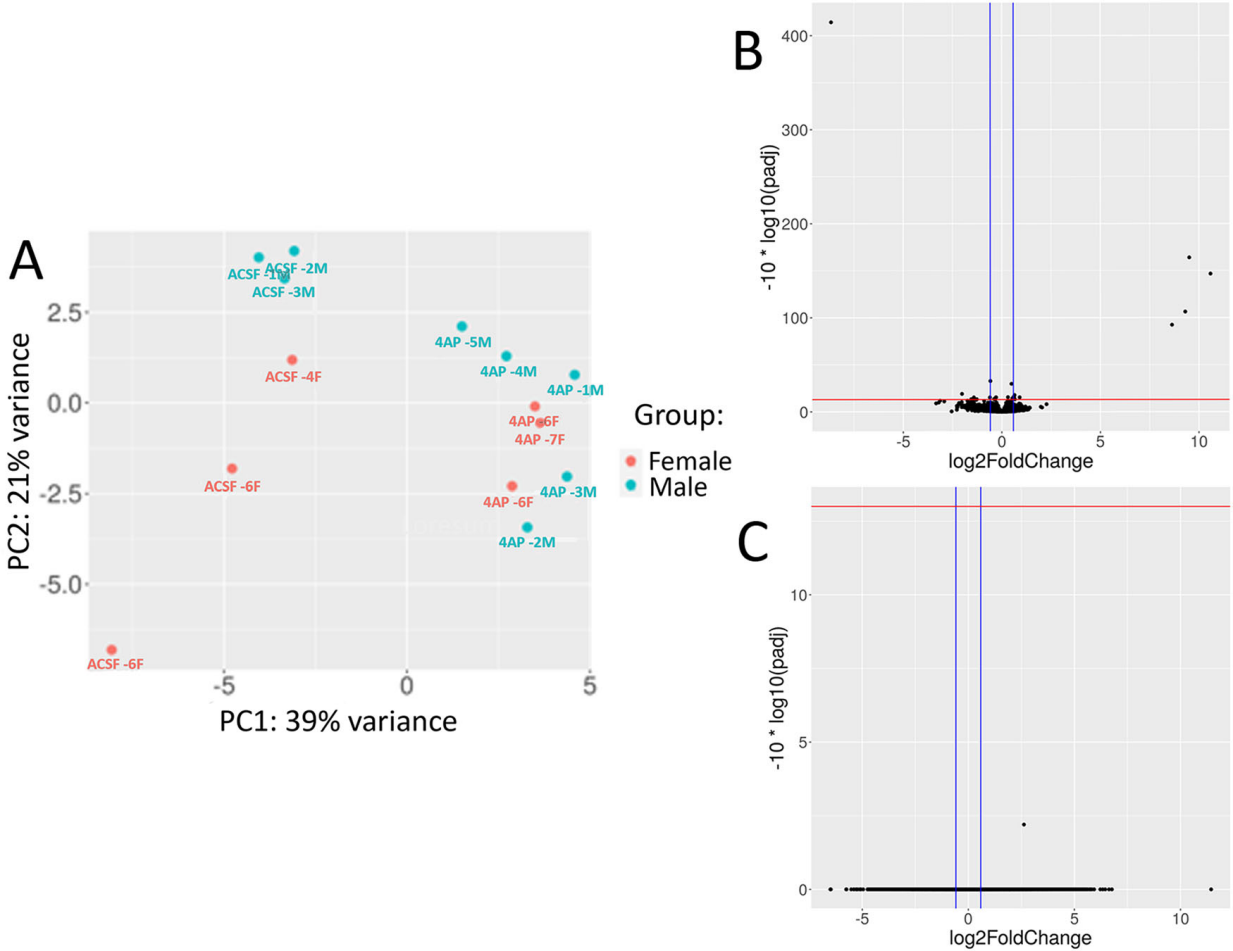
RNA-seq of male and female slices show no differences between sex in gene expression in response to epileptiform activity. ***A***, PCA plot of the RNA-seq gene expression data from each of the 14 samples separated by sex as shown by the female samples in orange and the males in blue. ***B***, Volcano plot showing DE of genes between male and female samples controlling for treatment condition. Upregulated genes are higher expressed in the male slices, and downregulated genes are present more in the female slices. ***C***, Volcano plot of genes in response to the combination of the sex of the sample and the seizure treatment. Genes are plotted with their log2FC against the −log10(*p*adj). The red horizontal line is at 13 which denotes a FDR = 0.05 and the blue vertical lines are at −0.58 and 0.58 showing the log2FC cutoffs.

To determine the specific factors contributing to the gene expression changes, namely, sex, seizure induction, or their combination, we employed the design feature of DESeq2 to account for these variables by segmenting the variance in the samples. Initially, we examined any sex-specific gene expression changes associated with epileptiform activity. Principal component analysis (PCA) revealed that both male and female samples exhibited the greatest variance between slices with induced seizure-like activity versus control samples, rather than being influenced by sex alone (Fig. 2*A*). Consistent with this result, only 15 genes exhibited DE based on sex of the sample, with the most significant difference between the sexes being found in genes on the X or Y chromosomes (Fig. 2*B*, Table 1). Further analysis of the combinatorial DE between the responses of each sex to seizures revealed no significant gene changes (Fig. 2*C*). Consequently, we concluded that induced seizure-like activity elicits minimal differences between male and female mice when utilizing an ex vivo preparation.

**Table 1.** List of differential expressed genes based on sex

Gene name	Log2FC	*p*adj	Chromosome	Description	Function
Eif2s3y	10.59003969	2.12 × 10^−15^	Y	Eukaryotic translation initiation factor 2, subunit 3, structural gene	Translational initiation
Ddx3y	9.512385256	3.98 × 10^−17^	Y	DEAD box helicase 3	Helicase activity
Kdm5d	9.308209984	2.30 × 10^−11^	Y	Lysine (K)-specific demethylase 5D	Chromatin demethylase
Xist	−8.653909241	3.78 × 10^−42^	X	Inactive X specific transcripts	No function
Uty	8.635518223	5.76 × 10^−10^	Y	Ubiquitously transcribed tetratricopeptide repeat containing	Chromatin binding
Depp1	−2.022647721	1.26 × 10^−2^	6	DEPP1 autophagy regulator	Autophagy
Tubb4b-ps2	−1.420474479	2.98 × 10^−2^	1	Tubulin, beta 4B class IVB, pseudogene 2	Tubulin
Etnppl	0.904044518	2.92 × 10^−2^	3	Ethanolamine phosphate phospholyase	Enzyme activity
Lrrc55	0.657426506	4.73 × 10^−2^	2	Leucine rich repeat containing 55	Ion transport
Cd34	0.641665353	1.67 × 10^−2^	1	CD34 antigen	Cell response to signals
Rskr	0.629185198	3.79 × 10^−2^	11	Ribosomal protein S6 kinase related	Protein phosphorylation
Rassf3	0.620279552	2.81 × 10^−2^	10	Ras association (RalGDS/AF-6) domain family member 3	Signal transduction
Crhbp	−0.60703997	3.00 × 10^−2^	13	Corticotropin releasing hormone binding protein	Peptide binding
Eng	0.587780636	2.81 × 10^−2^	2	Endoglin	Transforming growth factor beta activity
Eif2s3x	−0.580478737	5.00 × 10^−4^	X	Eukaryotic translation initiation factor 2, subunit 3, structural gene	Translational initiation

### DE analysis of ex vivo epileptiform activity reveals significant gene expression changes

**Figure 3.**
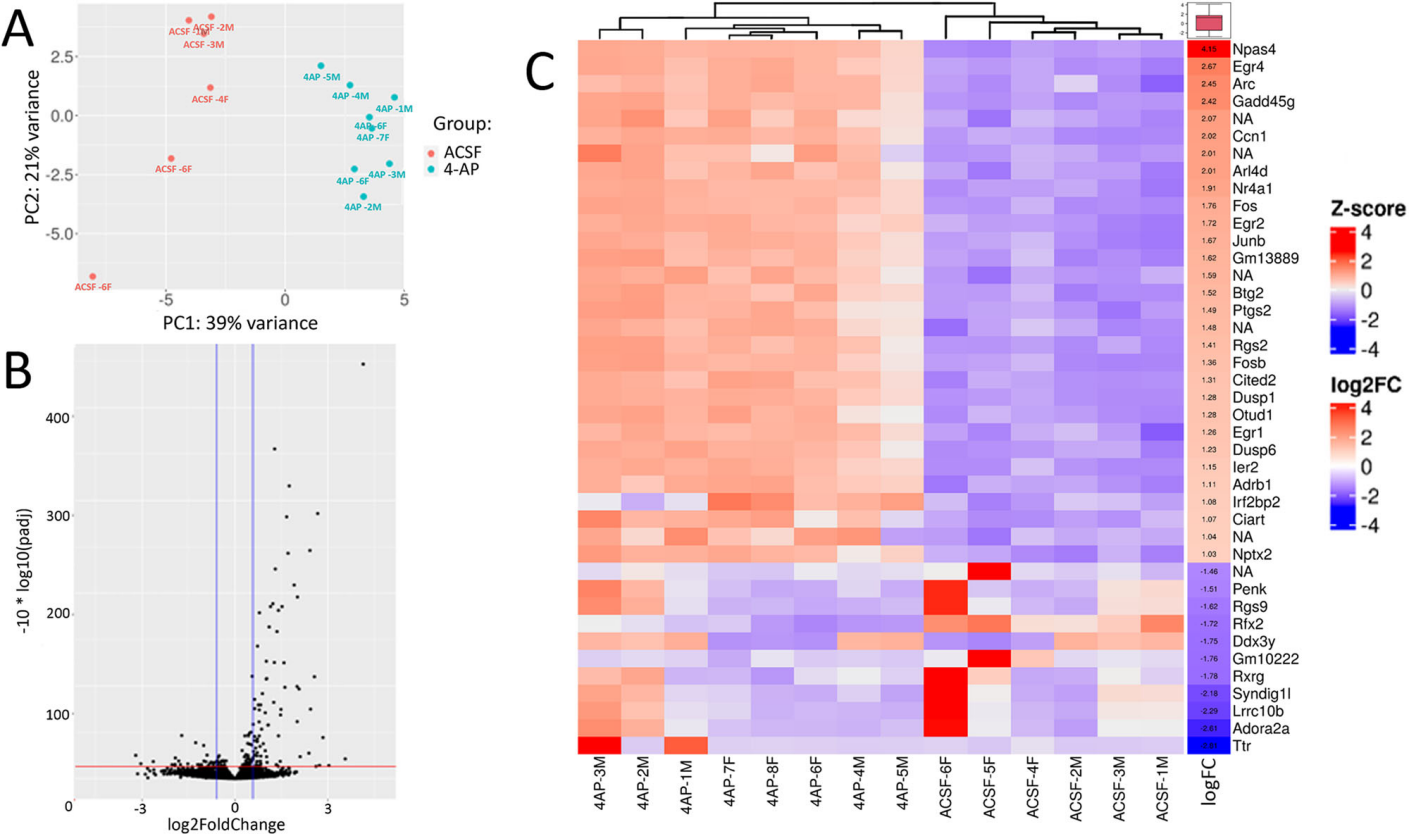
DE analysis of ex vivo slices shows large-scale gene expression changes in response to 4AP treatment. ***A***, PCA plot of the samples as in Figure 2*A*, separated by aCSF and 4AP treatment shown in orange and blue respectively. ***B***, Volcano plot as in Figure 2*B* of the DE genes after seizure induction. ***C***, Heatmap and hierarchical clustering of the top 30 upregulated and 10 downregulated genes for each of the samples shown in a column. A gradient from red to blue represents the expression of the gene in each sample, the red box denotes a higher expression, and a blue box denotes lower expression. The columns show the log2FC value and average expression values for the specific genes. See Extended Data Figure 3-1 for more details.

After finding no significant DE genes in our sex-specific analysis, we evaluated differences between the experimental groups, using both PCA and hierarchical clustering (Fig. 3*A*,*B*). The volcano plot in Figure 3*C* visually represents the DE genes, showing upregulation of 82 genes and downregulation of 28 genes in response to seizure induction (refer to Extended Data Fig. 3-1 for the full list). As expected, upon initial analysis of the DE genes, we observed that several of the most highly upregulated transcripts were immediate early genes, including c-Fos, early growth response protein 4 (*Egr4*), activity-regulated cytoskeleton-associated protein (*Arc*), ADP-ribosylation factor-like protein 4D (*Arl4d*), and neuronal PAS domain protein 4 (*Npas4*). Notably, *Npas4* has also been described as a transcription factor with a potential antiseizure role (D. Wang et al., 2014). Among the downregulated genes, we found examples that might be predicted to either decrease neuronal excitability (e.g., the calcium voltage channel Cav3.2, *Cacna1h*; log_2_FC of −0.70) or increase it (e.g., *Adora2a*, which codes for the adenosine A2A receptor; log_2_FC of −2.61).

10.1523/ENEURO.0520-23.2024.f3-1 Extended Data 3-1 Comprehensive list of DE genes. Download Extended Data 3-1, XLS file.

**Figure 4.**
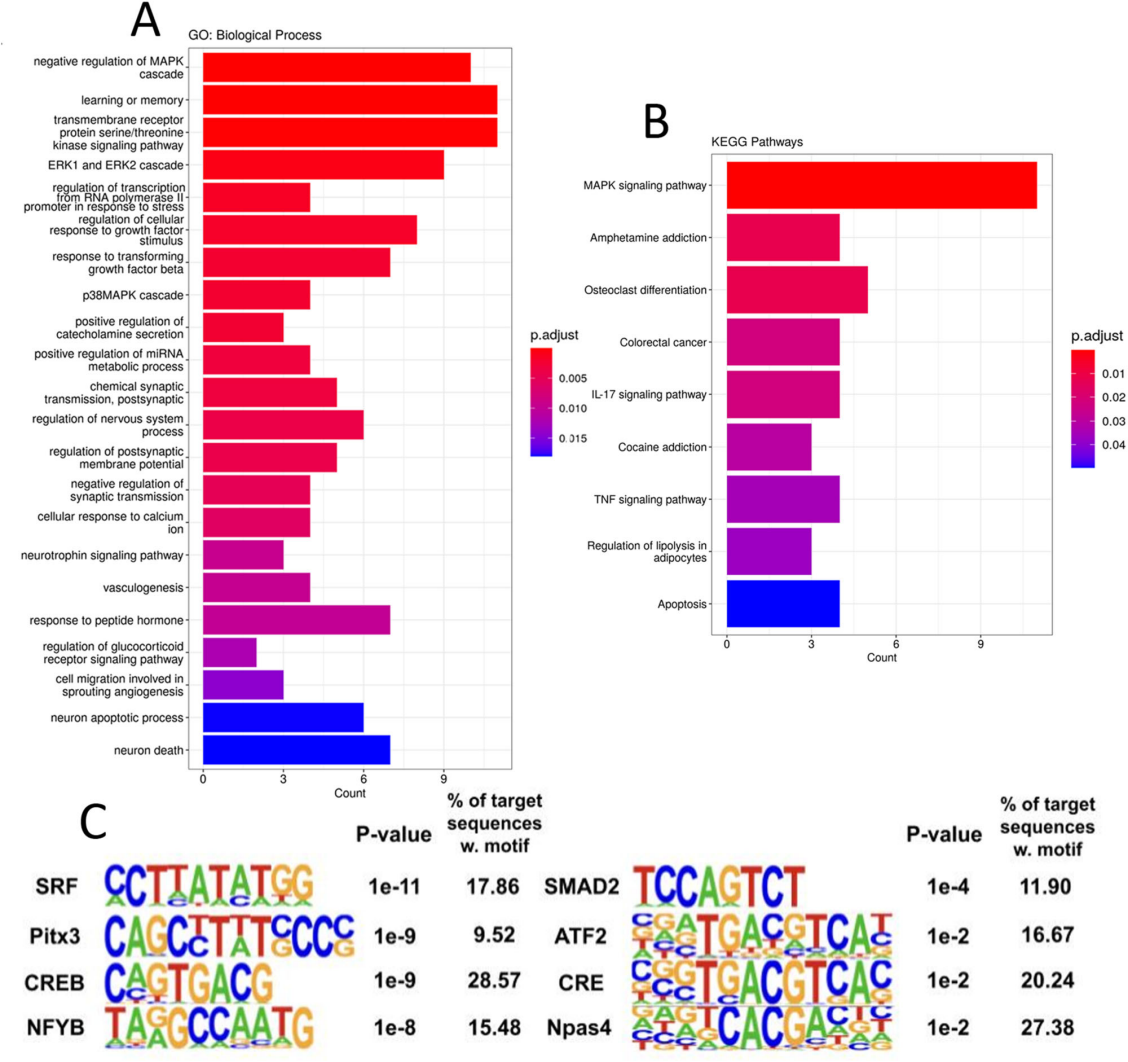
Gene ontology and pathway analysis reveal complex molecular regulatory networks post epileptiform activity. ***A***, Bar plot of 20 significantly enriched GO:Biological Process categories, bar color shows the relative significance based on the adjusted *p* value, and the length of the bar shows the number of upregulated genes enriched in the respective categories. ***B***, KEGG pathway enrichment analysis was performed on the gene set. Bar plot is arranged as in Figure 4*A* and includes the nine pathways that were significantly enriched. ***C***, Homer motif software results from enrichment analysis of the DE genes, motifs were either created de novo or were from the known database. *p* values were calculated against the full list of background genes in the software and % of genes with a respective transcription factor motif are shown. See Extended Data Figure 4-1 for more details.

In order to explore these gene expression changes more deeply, we used the Gene Ontology (GO) and KEGG pathway resources (Ashburner et al., 2000; Kanehisa and Goto, 2000; Gene Ontology Consortium et al., 2023). GO:Biological Process analysis revealed a significant enrichment of over 300 categories (refer to Extended Data Fig. 4-1 for the full list). To address redundancy resulting from hierarchical ontology parent–child relationships, we utilized the clusterProfiler package to streamline the GO results, resulting in 159 simplified categories. Figure 4*A* presents a selection of 20 enriched categories, illustrating various cellular responses to seizure activity. Among the enriched categories, several signaling pathways stood out, including the p38 MAPK, ERK 1/2 cascade, response to TGFB signaling, neurotropin signaling, calcium ion response, and response to peptide hormone pathways. While the involvement of the MAPK pathway and calcium ion response in epileptogenesis have long been recognized, the roles of these other pathways in relation to seizure disorders are not yet fully established (Steinlein, 2014; Gautam et al., 2021). Notably, several categories related to neuron death, apoptosis, and transcription activation in response to stress were also enriched, which may be attributed to the extensive brain damage caused by seizure activity. We found several intriguing pathways, including those involving the positive regulation of miRNAs, catecholamine secretion, and categories associated with vasculogenesis, angiogenesis, as well as learning and memory. Additionally, when we performed KEGG pathway enrichment analysis, we observed overlaps within the enriched pathways identified through GO analysis, particularly in the MAPK signaling pathway, TNF signaling pathway, and apoptosis (Fig. 4*B*). These findings provide valuable insights into the complex molecular processes underlying seizure-induced changes.

10.1523/ENEURO.0520-23.2024.f4-1 Extended Data 4-1 Comprehensive list containing GO:BP results and KEGG analysis. Download Extended Data 4-1, XLS file.

In light of the broad activity observed across various signaling pathways, we aimed to identify critical transcription factors responsible for the observed gene expression changes. To accomplish this, we analyzed our DE genes for enrichment of specific transcription factor binding motifs using the HOMER software. Of particular interest was the cAMP-response element binding protein (Creb), known to be activated by phosphorylation downstream of the MAPK and PKA pathways, as well as the crucial role of Creb-mediated transcriptional regulation in response to epileptogenesis (G. Wang et al., 2020; Mertz et al., 2020; Sun et al., 2022). Further analysis using HOMER showed a Creb motif upstream of 29% of the genes (Fig. 4*C*), indicative of the involvement of Creb in mediating a large proportion of our observed gene expression changes. Additionally, we identified highly enriched motifs for *Srf*, *Atf2*, and *Npas4*, which were upstream of over 15% of the DE genes. These transcription factors play significant roles in cellular stress response, and many have been implicated in mediating gene expression changes following seizures (Mielke et al., 1999; Losing et al., 2017; Shan et al., 2018). By identifying these enriched transcription factor binding motifs, we provide insights into the potential regulatory mechanisms driving the observed gene expression patterns. Further investigation into the specific interactions between these transcription factors and their target genes could shed light on the molecular processes underlying the cellular response to epileptiform activity.

**Figure 5.**
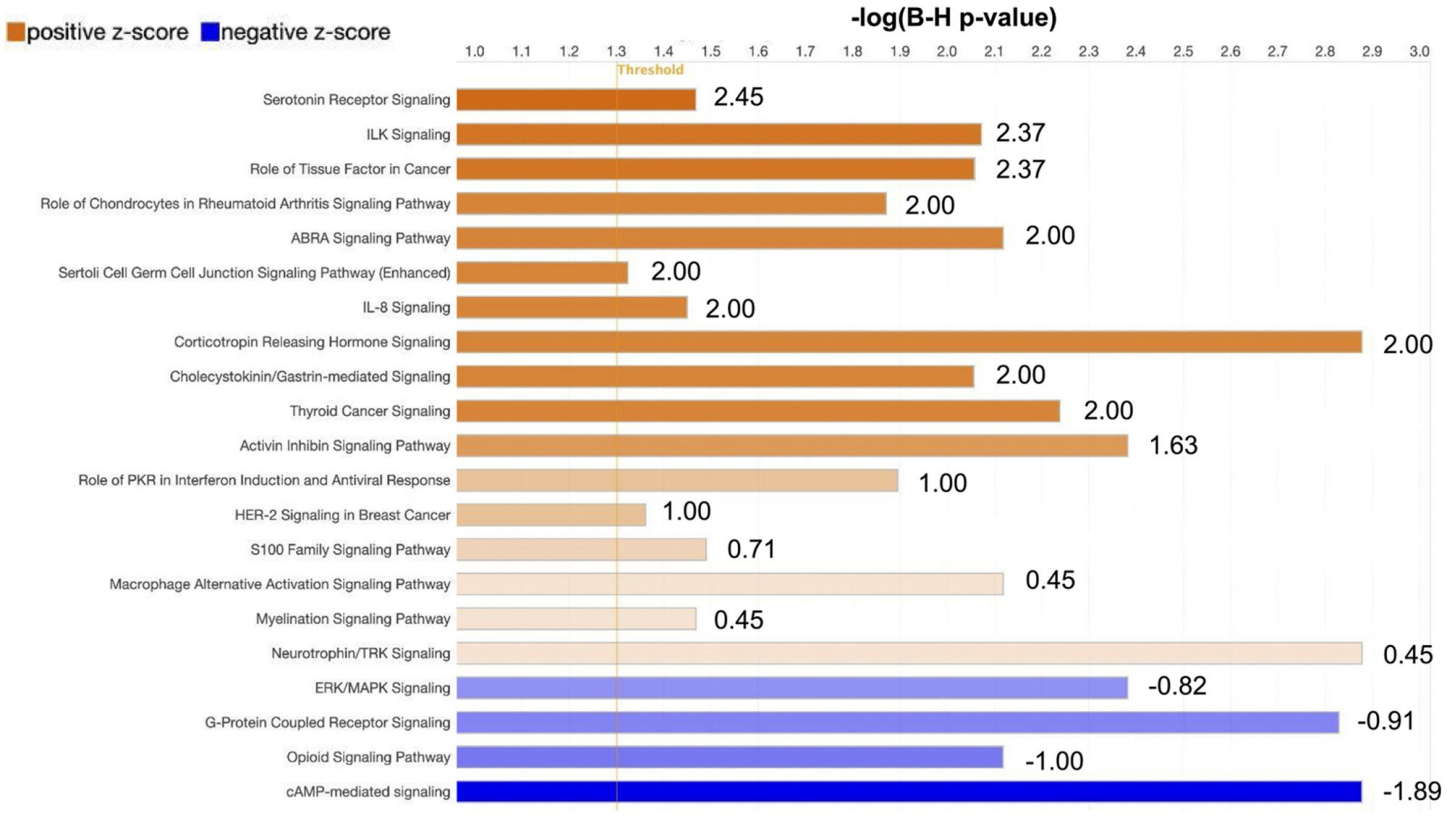
IPA predicts the activation of inflammatory regulatory networks and inhibition of cAMP signaling in response to epileptiform activity. Plot shows IPA canonical pathway prediction status based on the DE genes. Respective pathways are shown with the length of the bar representing the −log of the B-H adjusted *p* value for multiple testing. The bar color represents the *z*-score value activation (orange) or inhibition (blue) predicted state of the pathway. See Extended Data Figure 5-1 for more details.

To deepen our understanding of the specific cellular pathway responses to our observed gene expression changes, we leveraged Qiagen IPA analysis, which provides a machine learning algorithm and gene annotations to identify pathway enrichment and to predict pathway activation state (Kramer et al., 2014). Our results, shown in Figure 5, illustrate the complex signaling environment triggered by epileptiform activity. Several of the seizure-induced upregulated pathways that we identified are related to inflammatory and immune signaling (see Extended Data Fig. 5-1 for a comprehensive list), including IL-8 cytokine signaling, Hmgb1 production, neuroinflammation, and Cxcr4 signaling. Cxcr4, the receptor for the Cxcl12 ligand, has been implicated in neuroinflammation and has an emerging role in the induction of pro-seizure activity (Zhou et al., 2017). Furthermore, our GO and KEGG analysis also showed an enrichment for the production of IL-1β and IL-17 signaling, providing increased evidence of the importance of immune signaling after seizure activity (Dong et al., 2020; Wolinski et al., 2022). IPA predicted a downregulation of cAMP signaling, which would lead to a decrease in MAPK and PKA pathways (Bozzi et al., 2011). Upon further investigation, we observed an upregulation of several phosphatases involved in dampening MAPK signaling (Dusp1/4/5/6/14), many of which have been implicated in seizure responses (An et al., 2021). This might be considered to be a compensatory mechanism opposing the dramatic activation of these signaling pathways following seizure induction and could be potential therapeutic targets (Kirchner et al., 2020).

10.1523/ENEURO.0520-23.2024.f5-1 Extended Data 5-1 Comprehensive list of canonical pathways predicted to be activated or inhibited. Download Extended Data 5-1, XLS file.

### Ingenuity pathway analysis predicted epilepsy as the causal disease

**Figure 6.**
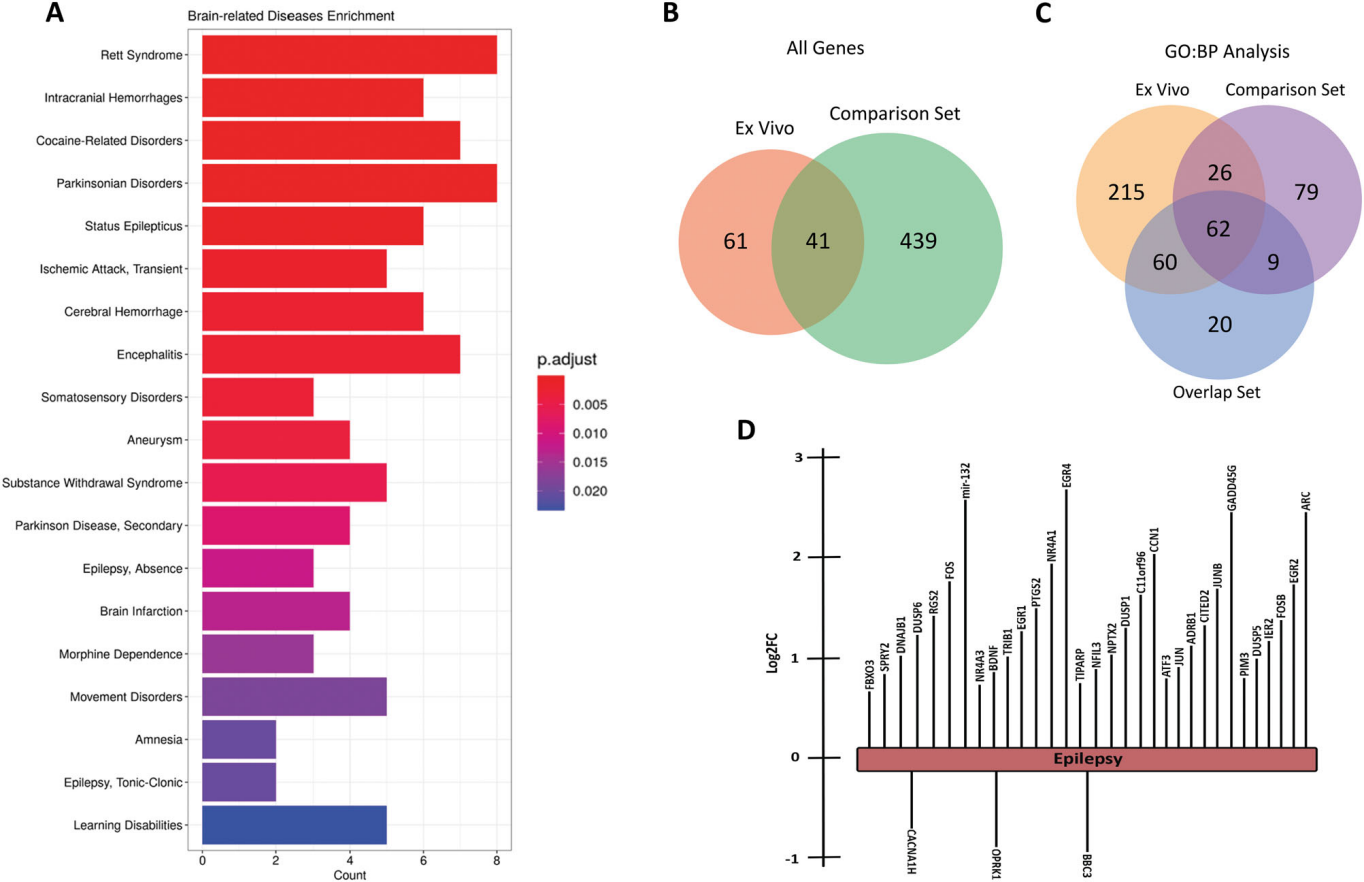
Disease Enrichment and IPA analysis of upregulated genes show significant links to epilepsy and other brain injuries. ***A***, Enrichment analysis of MeSH disease annotations from the upregulated gene set. A total of 247 disease terms were enriched and the bar plot is a selection of 20 enriched neuro-related diseases. Bar plot is as Figure 4. ***B***, Venn diagram of the overlap of the significantly DE genes from the CGS (Soon et al., 2023) and our ex vivo gene set. ***C***, GO:BP analysis was performed as described in Figure 4*A* on our ex vivo gene set, the comparison gene set, and the 41 genes that overlapped as shown in ***B***. The categories that were significantly enriched (adj. *p* value ≤0.05) were compared and plotted. ***D***, IPA analysis of gene set reveals epilepsy as the top disease state predicted from the DE genes with a *p* value of 1 × 10^−28^. Image shows the specific DE genes associated with epilepsy in the IPA knowledge base that were enriched in the IPA disease state analysis. Length of the bar represents the log2FC values for each gene. See Extended Data Figures 6-1, 6-2, and 6-3 for more details.

We next sought to determine whether the transcriptional changes following acute induction of seizure-like activity ex vivo could recapitulate gene expression changes established in previously published epilepsy models. To examine this, we utilized MeSH (Medical Subject Headings) analysis of disease-associated genes in published articles on PubMed (http://pubmed.ncbi.nlm.nih.gov). This analysis links terms, such as a particular gene, to specified categories, such as neurological conditions (Yu, 2018). Using MeSH, we performed an enrichment analysis comparing our DE gene set with disease terms. We identified 247 diseases that could potentially be associated with the observed gene expression changes, many of which were neurological disorders and brain injuries (Fig. 6*A*, complete list available in Extended Data Fig. 6-1). We found statistically significant enrichment for status epilepticus, absence epilepsy, and tonic–clonic epilepsy-related genes. Additionally, we observed categories related to substance addiction (e.g., cocaine-related disorders, morphine dependence), brain development disorders, and brain injuries.

10.1523/ENEURO.0520-23.2024.f6-1 Extended Data 6-1 Comprehensive list of diseases potentially associated with observed gene expression data. Download Extended Data 6-1, XLS file.

10.1523/ENEURO.0520-23.2024.f6-2 Extended Data 6-2 Comprehensive list of the Complete Gene Set from Soon et al. (2023) and list of overlapped genes. Download Extended Data 6-2, XLS file.

10.1523/ENEURO.0520-23.2024.f6-3 Extended Data 6-3 GO:BP analysis on the overlapping gene list, the comparison gene set, and our gene set. Download Extended Data 6-3, XLS file.

To further validate our ex vivo dataset, we searched the literature for other studies reporting RNA sequencing datasets from the acute stage of epileptogenesis. We focused on datasets that were comparable to our experimental design and technical approach to sequencing. We identified a study that investigated the transcriptomics of the early stage of seizure response using a pilocarpine-induced mouse model for 2 h before tissue isolation (Soon et al., 2023). While the Soon et al. (2023) study was narrowed to sequencing RNA from the nuclei of excitatory neurons from whole forebrain tissue samples, it represents an in vivo approach with a similar timeline following seizure induction as our dataset. The summarized results table from their DE analysis was obtained and we identified 480 differentially expressed genes (221 upregulated, 259 downregulated). This gene set, hereafter referred to as comparison gene set (CGS), can be found in Extended Data Figure 6-2. We found 41 genes that were codifferentially expressed in both our experiment and the Soon et al. (2023) study (Fig. 6*B*, complete gene list available in Extended Data Fig. 6-2).

To elucidate whether these overlapping genes are responsible for driving the signaling response postseizure induction or were simply experimental artifacts, we ran GO:BP analysis on both the overlapping gene list and the CGS and, along with our existing analysis of our gene set, we identified the shared statistically significant categories between these groups (full results can be found in Extended Data Fig. 6-3). Our results show that 62 categories were enriched in each of the analyses (Fig. 6*C*), suggesting that the 41 shared genes might recapitulate the functional output of seizure response and give further evidence to support our ex vivo results.

Given the technical differences between our experimental design and the Soon et al. (2023) study, we also ran ingenuity pathway analysis to determine the disease most likely responsible for our observed gene expression changes for further validation of our ex vivo approach. Epilepsy was the top disease state predicted based on the behavior of the 30 DE genes shown in Figure 6*D*. Our results across all three methods consistently suggest that our ex vivo approach can replicate the gene expression phenotype of seizure activity.

## Discussion

Ex vivo studies of large network activity patterns are sometimes disregarded because they are considered unrepresentative of the in vivo condition. Yet such simplified models offer many experimental advantages, and so it is helpful to examine how they might be used to tease apart the nature of especially complex neurological phenomena such as epileptic pathophysiology. Here, we performed transcriptomic analyses of brain slices that have experienced periods of intense epileptiform activation, finding changes in many genes that have been highlighted in other studies of epilepsy, as well as predicting key upstream transcription factors and potential therapeutic targets. Our ex vivo data were further validated by finding that the most closely related disorder associated with the gene expression patterns is indeed epilepsy.

These simplified, ex vivo preparations offer various benefits. Firstly, the ease of access to the tissue allows for a far tighter correlation between the observed gene expression profiles and specific patterns of electrophysiology activity, mapped with precision using multielectrode arrays (Mahadevan et al., 2022; Thouta et al., 2022), patch-clamp recordings, or imaging (Cammarota et al., 2013; Codadu et al., 2019b; Calin et al., 2021). Experimental manipulations using pharmacology, optogenetic, chemogenetic, or electrical stimulation can be similarly ​targeted (Calin et al., 2018; Papasavvas et al., 2020; Graham et al., 2023). These approaches allow for easy manipulation of both neuronal and non-neuronal cell classes, allowing for an increased ability to “dissect” the impact of individual cell classes on observed gene expression changes. Additionally, one animal can provide many experimental replicates, reducing variability and cost of the study, while also increasing the power of the study, thereby increasing the likelihood of identifying potential therapeutic targets. Ex vivo preparations do have their limitations; our preparations were fixed just a few hours after the slices are prepared, thus providing information only on the earliest transcriptional changes. Slower processes, though, may yet be amenable to experimental study in organotypic cultures (Lillis et al., 2015). In addition, ex vivo seizure induction is typically done with solution manipulations, such as the 4AP paradigm used in this study, which raises the question of whether the transcriptional changes observed were due to the drug treatment or the induced seizure activity. While our IPA analysis confirms our transcriptional changes largely overlap with expression profiles seen with human seizures and in in vivo rodent seizure models, these concerns can be addressed by future studies validating these results with other proconvulsant solutions, such as the low 0 Mg^2+^ paradigm (Parrish et al., 2019). In general, our validation of the patterns of transcriptional changes in these acute preparations, in conjunction with the advantages they offer, particularly with respect to animal welfare considerations (the “3Rs,” reduction, refinement, replacement; Lidster et al., 2016), provide a strong incentive for their greater adoption in the arsenal of experimental approaches for studying epilepsy.

Our ex vivo study has focused on assessment of total RNA transcriptional changes from neocortical tissue during acute seizure activity with whole tissue homogenates. We primarily focused on the neocortex due to the robustness of the presentation of tonic–clonic-like seizures that evolve into status epileptics-like discharges from these tissue preparations (Fig. 1). Other brain regions such as the hippocampus typically do not present tonic–clonic-like seizures from ex vivo preparations and instead display transient epileptiform discharges or do not have significant epileptiform activity until hours into the proconvulsant media (Codadu et al., 2019b). Future work could compare these two brain regions with the caveat that the epileptiform discharges develop with a different time scale and differ also with respect to the signature patterns of electrophysiological discharges. While our study focused on total RNA transcriptional changes, one could also use other techniques such as a ribosomal tag to “pull down” the mRNA that is primed for transcription and can allow for details of cell-type specific mRNA expression (Sanz et al., 2019). This would be a noteworthy follow-up experiment, where one can address transcriptional changes from mRNA being transcribed between different neuronal subtypes, glial cell types, and microglia. Indeed, our proof-of-principle study shapes the groundwork for future studies.

Notably, we found that slices from male and female mice show similar evolution of seizure-like activity following application of the convulsant drug 4AP, as well as similar patterns of seizure-induced gene expression changes. The absence of obvious sex differences in acutely induced seizure-like activity thus appears to be subtly different from the situation in chronic epilepsy. For instance, seizure severity has previously been reported to be higher in male than in female rats following pilocarpine administration (Matovu and Cavalheiro, 2022). In clinical medicine, men also show higher epilepsy prevalence while also having more severe seizures (Christensen et al., 2007; McHugh and Delanty, 2008; Christian et al., 2020), although a factor here may be that men are more likely to sustain head injuries. Notably, sex differences were found in a study of the gene expression profiles of blood samples from epilepsy patients from wide demographic range and multiple seizure types with epilepsy (Zhu et al., 2021). Further work will be required to ascertain whether gender differences are restricted to specific types of epilepsy or experimental models or are specific to the tissue sampled (blood vs the cortical tissue itself).

Since our own data found no difference between sexes, we pooled the male and female datasets for subsequent analyses to increase their statistical power, finding 82 genes that were upregulated and 28 downregulated, by the prior seizure-like activity. Many of these were immediate early genes, with the upregulation of Npas4 being particularly interesting given its suggested antiseizure role (D. Wang et al., 2014). Similarly, one may impute homeostatic, antiepileptic, negative feedback mechanisms (D. Wang et al., 2014; Y. Chen et al., 2014; Cain et al., 2018; Trevelyan et al., 2023) from the known function of certain genes (e.g., upregulation of *Npas4* or downregulation of *Cacna1h*). In contrast, other transcriptional changes we observed appear to enact positive feedback by promoting more seizure activity, such as the downregulation of *Adora2a*. Adenosine acts as an endogenous anticonvulsive, and the A2A receptor is one of its primary targets (Baltos et al., 2023). These results may be reconciled by the observation that long-term activation of the A2A receptor has been demonstrated to result in internalization (Palmer et al., 1994; Sheth et al., 2014), in which case downregulation of *Adora2a* may also be antiepileptic by the same mechanism.

We also examined gene changes within the context of their involvement in various signaling and cellular pathways. Notable among these were changes in transcription factors and genes involving the MAPK pathway, neurotrophins, and calcium ion response. Previous work also implicated the MAPK pathway as a potential disease-modifying route in epilepsy (Parrish et al., 2018), while transcription factors, such as Creb, are increasingly considered as targets for therapy in seizure disorders (Sun et al., 2022).

A major goal for the treatment of complex conditions such as epilepsy is to find how to use big transcriptional datasets for precision medicine for individual patients, but this still lies in the future (Orsini et al., 2018; Striano and Minassian, 2020; Knowles et al., 2022a). We present a case for progressing this field using simplified epilepsy models, such as those used here, paired with other new toolkits in neuroscience, such as optogenetics and chemogenetics, to advance our understanding of these conditions and create avenues for novel treatment options.

## Synthesis

Reviewing Editor: Harald Sontheimer, University of Virginia Decisions are customarily a result of the Reviewing Editor and the peer reviewers coming together and discussing their recommendations until a consensus is reached. When revisions are invited, a fact-based synthesis statement explaining their decision and outlining what is needed to prepare a revision will be listed below. The following reviewer(s) agreed to reveal their identity: Stefanie Robel, Michelle Olsen.

Both referees agree that the required revisions are

The current manuscript entitled 'RNA sequencing demonstrates ex vivo neocortical transcriptomic changes induced by epileptiform activity in male and female mice' has characterized transcriptional changes in gene expression in a newly developed an ex vivo seizure model. Here, over 100 genes were significantly differentially expressed following three hours of 4AP treatment in male and female neocortical tissue slices. This included a set of immediate early genes and several previously characterized epilepsy genes, including genes of the Mapk, Tnf and neuroinflammatory pathways. These findings are discussed in the context of the utility of simple model systems for understanding seizures and seizure induced transcriptional changes. Generally, this is a well-written, straightforward manuscript that provides acute seizure transcriptional data to the field. The ex-vivo model system provides a useful approach for comparison to other ex-vivo approaches, slice ephys, multi-electrode array, and optogenetic studies, and may be used as a resource along with in vivo preclinical model and human status epilepticus data for pharmaceutical target development. Limitations of the current article are detailed below.

Major

1) IPA analysis was useful for validating dataset was related to epilsepy, would also be useful to compare to with human or mouse status epilepticus transcriptional data set, at a minimum diagramming overlapping changing gene expression with another model system

2) No validation by qPCR of any of the targets

3) Limitations worth discussing -

a. utility of using a second pharmacological approach to induce seizures to delineate 4AP targets vs. general seizure driven transcripts

b. discuss why the focus on the cortex and not the hippocampus/entorhinal cortex? Would have been useful to compare cortex/hippocampus, are there region specific differences?

c. Given the experiments were evaluating acute response to 4AP, a ribotag approach, which evaluates dynamic regulation of gene expression would have been more relevant. For example, it is likely many more dynamically regulated genes were downregulated than identified because the investigator looking at total pool of existing RNAs.

4) As it relates to the point above, the investigators do not clarify if they performed RNA sequencing on total RNA or polyadenylated mRNA

5) Consider adding a paragraph to the introduction that discusses other cell populations in the CNS that are impacted by acute seizure activity. This may provide additional context as the experiments performed are in bulk tissue slices.

Minor editorial edits

• By convention, all mouse genes should have first letter capitalized, following letters lower case and italicized. When referring to mouse protein, it should be the same, but no italics

• Paragraph slice preparation - change word 'killed' to 'euthanized'

• Reference Liao, Smyth, and Shi 2019 missing from references section

• Cite reference/ for 100 uM 4AP to induces seizure activity

• Useful to add annotated function of each gene/protein in Table 1
